# Extended Treatment with Micron-Size Oral Palmitoylethanolamide (PEA) in Chronic Pain: A Systematic Review and Meta-Analysis

**DOI:** 10.3390/nu16111653

**Published:** 2024-05-28

**Authors:** Vittorio Schweiger, Carlo Schievano, Alvise Martini, Luca Polati, Giovanna Del Balzo, Salvatore Simari, Beatrice Milan, Gabriele Finco, Giustino Varrassi, Enrico Polati

**Affiliations:** 1 Department of Anesthesia, Intensive Care and Pain Therapy, Verona University Hospital, 37134 Verona, Italy; alvise.martini@univr.it (A.M.); pola.luca@hotmail.it (L.P.); salvatore.simari@aovr.veneto.it (S.S.); beatrice.milan@aovr.veneto.it (B.M.); enrico.polati@univr.it (E.P.); 2 Innovative Statistical Research, 35100 Padua, Italy; cs@i-stat.it; 3 Department of Medicine and Public Health, Section of Forensic Medicine, University of Verona, 37134 Verona, Italy; giovanna.delbalzo@univr.it; 4 Department of Medical Sciences and Public Health, University of Cagliari, 09042 Cagliari, Italy; gabriele.finco@unica.it; 5 Paolo Procacci Foundation, 00193 Rome, Italy; giuvarr@gmail.com

**Keywords:** palmitoylethanolamide, micron-size PEA, (ultra-)micronization, neuroinflammation, chronic pain, chronic pelvic pain

## Abstract

Palmitoylethanolamide (PEA) emerged over the years as a promising approach in the management of chronic pain. Despite the fact that the efficacy of micron-size PEA formulations appears to be time-dependent, the optimal timing has not yet been elucidated. This systematic review and meta-analysis aim to estimate the possible advantage of an extended treatment in the relief of chronic pain. The literature search was conducted consulting scientific databases, to identify clinical trials in which micron-size PEA was administered for at least 60 days, and pain assessed by the Visual Analogue Scale (VAS) or Numeric Rating Scale (NRS). Nine studies matched the required criteria, for a total of 742 patients involved. The meta-analysis showed a statistically and clinically significant pain intensity reduction after 60 days of micron-size PEA supplementation, compared to 30 days (1.36 points, *p* < 0.01). The secondary analysis revealed a weighted NRS/VAS score decrease of 2.08 points within the first month of treatment. These two obtained scores corresponded to a 35.1% pain intensity reduction within the first month, followed by a further 35.4% during the second month. Overall, these results confirm the clinically relevant and time-depended pain-relieving effect of micron-size PEA and therefore the advantage of an extended treatment, especially in patient with incomplete pain management.

## 1. Introduction

According to the International Association for the Study of Pain (IASP), chronic pain is defined as “pain that persists or recurs for longer than three months” [1]. It affects between a third and half of the population worldwide [2]. Complete relief from chronic pain is often unrealistic and integrated pain management using multimodal therapies is currently the advocated strategy [3]. These therapies may include exercise, physical therapy, massage, cognitive and behavioral therapy, mind–body practices, neuroablative or neuromodulative treatments and drugs [4]. To date, the pharmacological options for analgesia are limited to opioids, nonsteroidal anti-inflammatory drugs (NSAIDs), acetaminophen, antidepressants and anticonvulsants, which provide variable efficacy and common adverse events (AEs) [5]. A growing body of evidence suggests that neuroinflammation plays an important role in the induction and maintenance of chronic pain [6]. In response to peripheral and central injury, glial cells, in particular microglia, show an increased activity in several pain pathways. After trauma or nerve injury, microglia increase the expression of markers and receptors, and release inflammatory mediators that contribute to neuronal excitability and pain generation [7]. Microglia also respond to inflammatory signals coming from mast cells, whose activation can modify the sensory transmission via a wide spectrum of pain mediators which can directly interact with sensory nerve terminals [7,8,9]. Sensory neurons, by releasing neuropeptides, may in turn increase mast cells activation/degranulation, which results in nociceptor sensitization, reduced pain threshold at the site of inflammation, dysfunctional pain signaling and hyperalgesia [10]. The involvement of mast cells and microglia in the development of hyperalgesia has been already demonstrated in different conditions such as chronic low back pain, visceral or pelvic pain, migraine, trigemino-cervical pain, lumbosacral pain and neurological disorders like Parkinson’s disease, multiple sclerosis and stroke [11,12,13].

While the excess of peripheral nociceptive stimulation leads to peripheral sensitization, which is essential for the development of pain, chronic pain is the result of neuronal plasticity and the so called “central sensitization”. This phenomenon derives from multiple and complex interactions between the nervous system, the immune system cells and other non-neuronal and non-immune cells, which play a fundamental role in both the amplification and chronicization of pain, also potentially influencing its different emotional and cognitive components [14]. Once central sensitization occurs, treatments for the associated syndromes require a multimodal approach that includes physical therapy, behavioral pain psychology and pharmacological agents specifically targeting neuroinflammation, pain modulation and pain pathways amplification [15]. Based on this evidence, the modulation of neuroinflammation emerged as an efficacious therapeutic option for pain control [16]. Among neuromodulators, the endogenous lipid mediator palmitoylethanolamide (PEA), a member of the *N*-acylethanolamine family, naturally present in several food sources, has been reported to exert anti-allodynic and anti-hyperalgesic effects by down-modulating mast cell activation and controlling glial cell behaviors [9,10]. These immuno-regulatory and analgesic properties of PEA are mediated through direct and indirect biological pathways, involving cannabinoid receptors type 1 (CB1) and type 2 (CB2), cannabinoid-related G protein-coupled receptors 55 (GPR55) and 19 (GPR119), transient receptor potential vanilloid type 1 (TRPV1) channels, and nuclear peroxisome proliferator-activated receptor-alpha (PPAR-a) [17,18]. PEA is endogenously produced in all tissues of the human body in response to stressful conditions, inflammatory stimuli, injury or pain [19]. When these conditions are protracted, PEA may be depleted and exogenous PEA administration may become important to restore its protective, anti-inflammatory and analgesic effects [20]. Although PEA is present in several dietary sources, its levels in foods are too low to represent an adequate intake in pathological conditions and a further exogenous supplementation must be considered [21,22,23]. However, exogenous PEA administration may significantly counteract neuroinflammation at the cellular level only in micronized (mPEA, 2–10 μm range) or ultra-micronized (umPEA, 0.8–6 μm range) forms, while in the native state (naïve PEA), due to the large particle size (from 100 up to 2000 μm range), PEA showed a poor absorption that significantly reduced its distribution and bioavailability, providing a low biological effect [23,24,25,26]. Also, it was demonstrated that mPEA and umPEA, in combination with natural compounds (co-micronized or co-ultra-micronized forms, respectively), such as the antioxidant polydatin (e.g., mPEAPol), demonstrated a synergistic effects and stronger biological activity [21]. From a clinical perspective, many publications reported the efficacy of all these micron-size PEA formulations (micronized, ultra-micronized, co-micronized and co-ultra-micronized) in chronic pain conditions of different etiologies [16,27,28,29,30,31,32,33,34,35]. Furthermore, several clinical trials have highlighted the time-dependent efficacy of these preparations in the management of chronic pain [36,37,38,39,40,41,42,43,44,45,46,47,48,49,50,51,52,53,54,55], but none of them have quantified the advantage of extending the supplementation beyond the first month of treatment. The aim of this systematic review and meta-analysis was therefore to estimate this improvement in different chronic pain conditions. 

## 2. Materials and Methods

### 2.1. Search Strategy

This review adhered to the Preferred Reporting Items for Systematic Review and Meta-analysis (PRISMA) guidelines [56]. The search technique was performed according to the PICO (Population/Participants, Interventions, Comparisons, Outcome) approach. We searched in the PubMed and Scopus databases until September 2023 using the following keywords: “micronized Palmitoylethanolamide” or “ultra-micronized Palmitoylethanolamide” AND “chronic pain” or “chronic pelvic pain” or “neuropathic pain” or “neuropathy” or “neuralgia”. A supplementary search examined the references of the papers selected after the initial search. No restrictions regarding the publication date or language were imposed.

### 2.2. Inclusion and Exclusion Criteria

We searched studies including patients of both genders with different ages and chronic pain of various etiologies, submitted to different preparations of micron-size PEA alone or as an add-on to treatment with other pain medications. The eligible studies had to include: (i) pain intensity measurement using either the Visual Analogue Scale (VAS, 0–100 mm) or the Numeric Rating Scale (NRS, 0–10 points) and (ii) the assessment of pain at baseline, 30 days and 60 days after the start of micron-size PEA’s administration. 

Narrative or systematic reviews, meta-analyses, book chapters, abstracts, congress communications and case reports were excluded from the analysis. 

The initial search results underwent a thorough deduplication process, wherein two authors separately examined the titles and abstracts to identify and exclude papers that were not relevant. Afterward, the entire text was scrutinized to determine whether items met the qualifying criteria. The authors of three publications were contacted and requested to deliver additional data relevant for the analysis and not reported in the published papers [53,54,55].

### 2.3. Endpoints

The primary endpoint was the assessment of the further change in pain intensity, using VAS or NRS scores, after two months of micron-size PEA administration, compared to a 30-day treatment period (T30–T60 interval). The secondary endpoints were the assessment of the weighted change in pain scores from baseline to the first month (T0–T30), and the percentage changes in NRS/VAS scores during the two different intervals T0–T30 and T30–T60.

### 2.4. Risk of Bias

The evaluation of the potential for bias in each study included in the meta-analysis was performed autonomously by two authors. The Cochrane risk of bias (RoB) instrument, RoB2, was used to assess the quality of randomized clinical trials [57]. The Risk of Bias in non-randomized studies of Interventions (ROBINS-I) tool was adopted for non-randomized studies [58]. The evaluation covered different areas (5 areas for randomized trials and 7 for non-randomized studies) that addressed potential biases related to the randomization process (RoB D1), confounding (ROBINS-I D1), participant selection (ROBINS-I D2), intervention classification (ROBINS-I D3), deviation from intended interventions (RoB D2 and ROBINS-I D4), missing data (RoB D3 and ROBINS-I D5), outcome measurement (RoB D4 and ROBINS-I D6) and selection of reported results (RoB D5 and ROBINS-I D7). These evaluations classify each study’s risk of bias as low, moderate, serious, critical or with some concerns. The RoB assessment visualization was created using the Cochrane Risk-of-bias visualization (Robvis) program [59].

### 2.5. Statistical Analysis

A random effects model, which handles the non-homogeneity among the selected studies, was employed to estimate the primary efficacy outcome [60]. In instances wherein studies reported multiple interventional groups, the outcomes of each group were treated as independent entities. In such cases where the standard error of the mean (SEM) was presented, the standard deviation (SD) was computed using the relevant mathematical formula. When the SD of the NRS/VAS score change from the 30-day endpoint to the T60 final follow-up was not provided in the original articles, a conservative repeated measures correlation of 0.4 was employed for calculation [61], with data from the study by Schweiger et al. [49] utilized to validate this choice, resulting in a repeated measures correlation of 0.43. The average pain intensity change (estimated effect) and its corresponding 95% confidence interval (CI) were computed for each study. Heterogeneity among studies was assessed using *I*^2^ and τ^2^ statistics. The meta-analysis was executed using R 4.3.1 software, with statistical significance set at *p* < 0.05. The weighted average pain intensity score at each evaluation time point was calculated for secondary outcomes, considering the weight assigned to each study resulting from the meta-analysis. The corresponding SEM was determined from the SD by the software. The treatment effect was expressed as the change in the weighted average NRS/VAS score between baseline, T30 and T60 endpoints (T30 vs. T0, T60 vs. T0, T60 vs. T30). The change in pain intensity between T0–T30 and T30–T60, computed as a percentage, served to estimate the effect at each follow-up relative to the preceding one (T30 vs. T0, T60 vs. T30) and, in comparison to the overall impact (T60–T0) achieved during the course of the studies.

## 3. Results

### 3.1. Search Results

**Figure 1:**
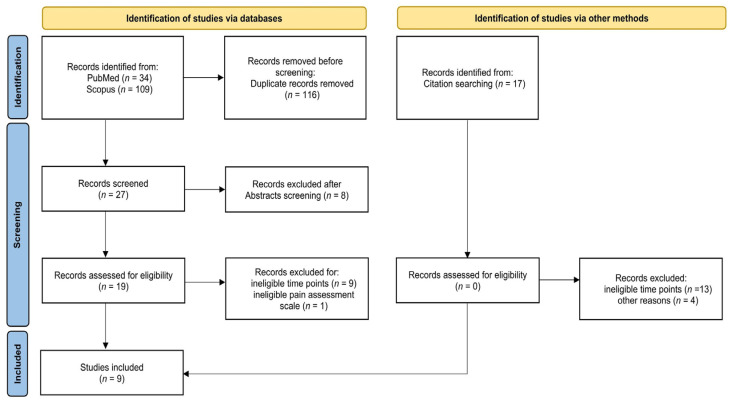
Flowchart of the search strategy and studies selection according to PRISMA [62].

A total of 160 studies were identified from the initial search. Among these, 116 studies were excluded after screening and removing duplications. Then, 44 studies were assessed for eligibility. After abstract screening, eight studies were excluded, and 36 studies were analyzed to evaluate the required criteria. Of these, nine studies were deemed eligible and included in the systematic review [40,42,49,50,51,52,53,54,55]. The flowchart of the study selection process is reported in Figure 1.

### 3.2. Description of the Included Studies 

Out of the nine selected studies, one is a randomized clinical trial [53], while eight are uncontrolled studies with various designs (observational prospective or retrospective, pilot studies or case series) [40,42,49,50,51,52,54,55]. Five studies focused on chronic pelvic pain [40,52,53,54,55], three on chronic low back pain of different origins [i.e., nonsurgical lumbar radiculopathies [50], FBSS (failed back surgery syndrome, to date modified as persistent spinal pain syndrome, PSPS) [51], lumbosciatica [42] and one study focused on chronic pain related to fibromyalgia syndrome (FMS) [49]. The selected studies involved a total of 742 patients. Among these, 614 patients were treated with umPEA (NORMAST^®^600), 81 with mPEAPol (PELVILEN^®^), 30 with mPEAPol + umPEA (PELVILEN^®^ DUAL ACT) and 17 with a combined treatment started with umPEA (NORMAST^®^600) and followed by mPEAPol (PELVILEN^®^). All these preparations are released on the market by a single manufacturing company (Epitech Group SpA, Saccolongo, Padova, Italy). The dosages of these preparations, generally used as an add-on supplement to pain medications, ranged from 600 mg to 1800 mg per day for umPEA and from 800 mg + 80 mg to 1200 mg + 120 mg per day for mPEAPol. UmPEA was generally prescribed twice daily during the first period of treatment, followed by once a day until the final follow-up [42,49,50,51,55], whereas mPEAPol and the mPEAPol + umPEA combination were administered twice daily for the entire study duration [40,52,53,54,55]. Pain intensity was assessed using the VAS score in six studies (66.7%) [40,49,50,51,52,55] and the NRS score in three studies (33.3%) [42,53,54]. Mild AEs, mainly of gastrointestinal type, were reported in only three studies and generally judged by the authors as unrelated to the treatment [40,49,54]. However, in all the included studies, these micron-size formulations have been considered safe and with a significant tolerability profile, and no pharmacological interaction with the concomitant analgesic and/or anti-inflammatory therapies were reported. The main characteristics of the included studies are presented in Table 1.

### 3.3. Risk of Bias

**Figure 2:**
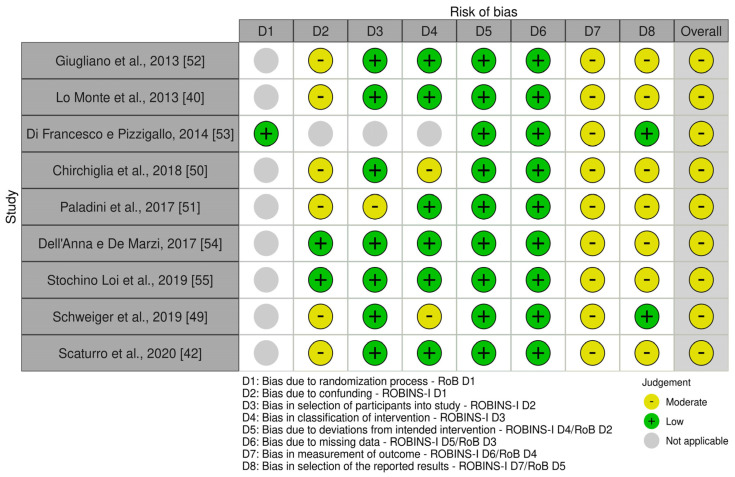
Robvis traffic light plot for the risk of bias of the studies included in the meta-analysis [40,42,49,50,51,52,53,54,55].

**Figure 3:**
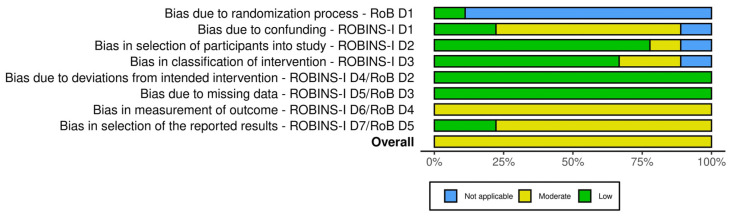
Robvis summary plot for the risk of bias of the studies included in the meta-analysis.

All studies were identified as having a moderate risk of bias, since they all showed moderate bias in the outcome measurement domain. Six trials exhibited bias attributable to confounding factors [40,42,49,50,51,52], one in participant selection [51] and two in the classification of intervention [49,50]. In all studies, the bias related to deviations from the intended intervention as well as to missing data was deemed low. Concerns about the selection of reported results were rated at a moderate risk for most of the studies. No sensitivity analysis was performed since none of the studies had a high risk of bias. The ROB summary is presented in Figure 2 and Figure 3.

### 3.4. Primary Endpoint: T60 vs. T30 Pain Intensity Change 

**Figure 4:**
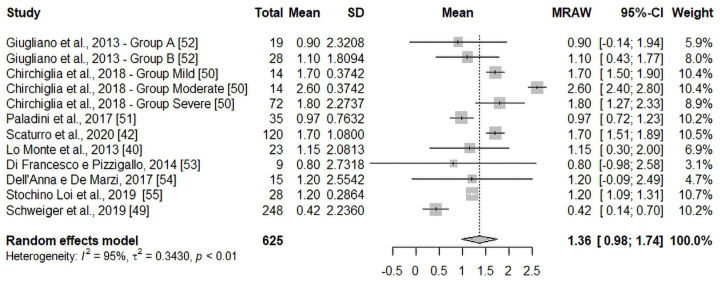
Forest plot of the effect of a 60-day supplementation with micron-size PEA on chronic pain intensity reduction. Positive mean values (>0) represent the additional effect after the first 30 days of supplementation. Squares display the estimate impact, the size of each square reflects the weight assigned to each study. Horizontal lines represent the 95% CI for each estimate effect. The diamond width, which indicates the total 95% CI, represents the overall effect of intervention estimate using a random statistical model. *I*^2^ and τ^2^ statistics measure the heterogeneity [40,42,49,50,51,52,53,54,55].

Regarding the primary endpoint, the meta-analysis showed that the extension of micron-size PEA supplementation to 60 days led to a further pain reduction effect compared to the first 30 days, therefore favoring the 60-day intervention. The estimate average pain intensity reduction was of 1.36 points (95% CI: 0.98; 1.74; *p* < 0.01) on the NRS/VAS scale between T30 and T60. This overall effect was statistically significant, although the heterogeneity among the included studies was high (*I*^2^ = 95%; τ^2^ = 0.34; *p* < 0.01). The forest plot of the meta-analysis is presented in Figure 4.

### 3.5. Secondary Endpoints: T30 vs. T0 Pain Intensity Change, and Percentage Variation over Time

Table 2 shows the number of patients and the average pain intensity scores at T0, T30 and T60 for each study considered in the meta-analysis.

**Figure 5:**
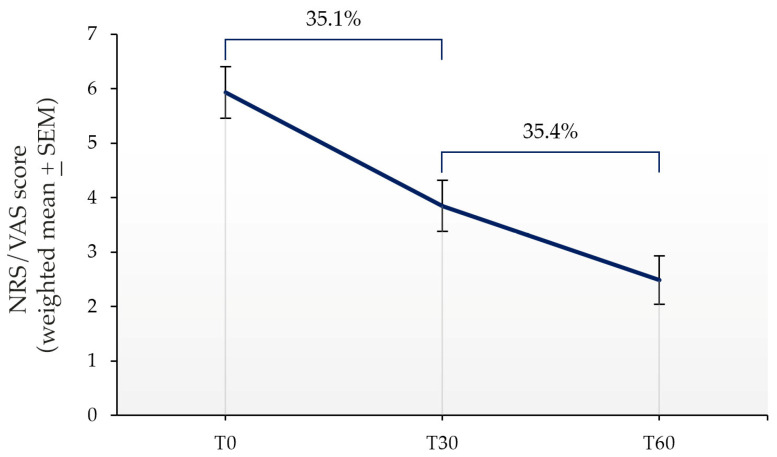
NRS/VAS score improvement during the first and the second month of micron-size PEA treatment and the corresponding percentage of pain intensity reduction after each time point compared to the previous one.

Regarding the secondary endpoints, the data analysis showed a weighted reduction of 2.08 points from baseline to the first month (T30). This reduction was equivalent to a decrease of 35.1% in the NRS/VAS score during the first 30 days of treatment (T0–T30 interval). The decrease of 1.36 points, highlighted during the second month (T30–T60 period) by the meta-analysis, corresponded to a further reduction of 35.4% in pain intensity (Table 3 and Figure 5). Comparing the reduction in pain score obtained during the T0–T30 and T30–T60 intervals with the overall improvement observed during the 2-month period (T0–T60, 3.44 points, 100%), the NRS/VAS score reduction was of 60.4% after the first month and of 39.6% after 60 days of micron-size PEA supplementation (Table 3 and Figure 5).

## 4. Discussion

To date, several clinical studies have demonstrated the role of oral PEA in its micron-size formulations in the management of various painful conditions, with an efficacy that appears to be time-dependent [36,37,38,39,40,41,42,43,44,45,46,47,48,49,50,51,52,53,54,55]. Also, this time-dependency effect was highlighted in a recent meta-analysis, suggesting a treatment of at least four weeks with PEA [63]. Despite this evidence, the optimal timing and treatment duration have not been clearly elucidated or explored in detail until now. Our systematic review and meta-analysis, focused primarily on whether an extended supplementation with micron-size oral PEA could lead to additional pain relief, showed a statistically significant efficacy of this supplementation after 60 days compared to the 30-day treatment. The decrease in pain intensity was also accompanied by an improvement in QoL, without relevant AEs or pharmacological interaction with the concomitant analgesic therapies during the entire considered period. Furthermore, in two out the nine considered studies, the improvement in pain intensity was accompanied by a reduced need of medications, suggesting that the supplementation with oral micron-size PEA may have an analgesics’ sparing effect, reducing also the incidence of drugs-related AEs [40,55]. From a pathophysiological point of view, the benefits of an extended treatment with oral micron-size PEA in chronic pain may be explained by considering the specific role of the neuroinflammation in promoting and maintaining pain in different conditions. It is well known that neuroinflammation plays a key role in the onset and evolution of chronic pain and probably also in the transition from acute to chronic pain [64]. In fact, the so-called “non-neuronal cells” (microglia and mast cells) are considered the main responsible actors of this process [10]. Their prolonged activation leads to the uncontrolled release of pro-inflammatory mediators, resulting in changes in pain signaling pathways and chronic pain development [65,66]. The antinociceptive properties of oral PEA mainly depend on the down-regulation of these non-neuronal cells, through direct and indirect mechanisms [23]. It is also established that the timing of intervention is crucial in such situations and that early treatment is therefore essential [67,68]. 

The secondary analysis conducted in this work made it possible to estimate a weighted decrease of 2.08 points in the NRS/VAS score between the initial measurement and the 30-day follow-up, followed by a further decrease of 1.36 points in pain scores during the second month (T30–T60 interval). These results are consistent with the literature observations of the time-dependent effectiveness of micron-size PEA, as previously reported. However, one may argue that the reduction in small points in pain scores, while statistically significant, may not be clinically relevant for the patient. Although the clinically significant change in pain intensity measured by the scales or the minimum percentage of pain reduction from baseline was not clearly defined in the literature, some data are available in this context. Farrar et al., evaluating data from 2724 patients suffering from chronic pain of different etiologies and enrolled in 10 clinical trials, estimated that a reduction of approximately 2 points or of approximately 30% in the NRS score represent a clinically relevant difference in patients’ QoL [69]. The authors also demonstrated that, in studies with greater variability in baseline pain, such as those with no minimum baseline pain requirements, clinical relevance should be defined in terms of percent change, as the relationship between the percentage change and the patient’s impression of the change should be more coherent [69]. Furthermore, in some diseases like fibromyalgia syndrome, a limited improvement in perceived pain or QoL was also deemed by the authors to be clinically relevant, particularly in a patients’ population that has a very poor response to available conventional treatments [49,70]. Based on these findings, since in only four of the nine included studies the requirement for patient eligibility was an NRS/VAS score ≥ 5, to define whether the change in pain intensity was clinically relevant, the percentage reduction in the NRS/VAS score was also considered in our analysis. A pain intensity improvement of 35.1% was obtained during the first 30 days of micron-size PEA treatment, and a further 35.4% improvement was achieved during the second month, compared to the previous one, demonstrating that the supplementation with oral micron-size PEA allowed for a clinically significant and continued pain reduction over time. 

Our analysis has some limitations, the main one of which is the uncontrolled nature of the included studies. Unfortunately, only a relatively small number of publications met the strict inclusion criteria, and all were inherently biased. Several studies considered just a few patients, who in general also underwent different concomitant therapies. In addition, the meta-analysis revealed a high heterogeneity of the selected studies, probably due to the different pathological conditions and/or to the difference in the oral micron-size PEA daily dosage. Taken together, all these factors may limit the statistical power of the final result.

## 5. Conclusions

This meta-analysis supports the advantage of extending the supplementation with oral micron-size PEA beyond the first month of treatment and the overall beneficial effect of this supplementation in the management of chronic pain. Micron-size PEA could therefore represent a possible adjunctive approach, with a noticeable tolerability profile, for patients experiencing pain associated with chronic diseases and already undergoing debilitating pharmacological therapies. However, due to the limitations of the analysis, more in-depth methodological studies are needed to corroborate these observations. 

## Figures and Tables

**Table 1:** Study characteristics.

Reference	Study Design	Number of Patients	Chronic Pain Condition	Pain Tool	Treatment	Concomitant Therapies	Main Results
Chirchiglia et al., 2018 [50]	Retrospective, case-series	100 (73 males, 27 females)	Low back pain (nonsurgical lumbar radiculopathies with or without sciatica)	VAS	1st cycle: 1200 mg/day sublingual **umPEA** for 10 days; 1200 mg/day umPEA tablets for 20 days 2nd cycle: 600 mg/day umPEA tablets for 1 month	1st cycle: Paracetamol 500 mg + codeine 30 mg/day for 4 days, then for 1 month as needed 2nd cycle: Paracetamol 500 mg + codeine 30 mg/day as needed	Significant decrease in pain intensity after one month of treatment with a further improvement after the second cycle. Greater effect on patients with mild and moderate pain
Paladini et al., 2017 [51]	Observational, open-label	35 (15 males, 20 females)	Low back pain (failed back surgery syndrome)	VAS	1200 mg/day **umPEA** for 1 month; subsequently 600 mg/day for the second month	Tapentadol 150 mg/day Pregabalin 300 mg/day	Further and significant decrease in pain intensity after umPEA supplementation
Scaturro et al., 2020 [42]	Prospective, observational	120 (37 males, 83 females)	Low back pain (lumbosciatica/ lumbocruralgia)	NRS	1200 mg/day **umPEA** for 20 days; subsequently 600 mg/day for 40 days	Pregabalin 150 mg/day or oxycodone 10 mg/day, daily functional rehabilitation, relaxation massage	Significant reduction in chronic pain to a not clinically relevant intensity; improvement in QoL; decrease in pain-dependent disability
Lo Monte et al., 2013 [40]	Pilot, open-label	24 females	Chronic pelvic pain (endometriosis)	VAS	800 + 80 mg/day **mPEAPol** for 90 days	Analgesics and hormonal therapies	Significant reduction in pelvic pain, dysmenorrhea and dyspareunia; improvement in QoL; decrease NSAIDs consumption
Giugliano et al., 2013 [52]	Prospective, open-label	47 females *Group A*: 19 recto-vaginal endometriosis; *Group B*: 28 ovarian endometriosis	Chronic pelvic pain (endometriosis)	VAS	800 + 80 mg/day **mPEAPol** for 90 days	Estrogen–progestin pill (13 patients group A, 18 patients group B), anti-inflammatory drugs (6 patients group A, 10 patients group B)	Significant reduction in pelvic pain, dysmenorrhea, dyspareunia regardless of lesion site already after 30 days, reaching the maximum relief after 60 days
Di Francesco and Pizzigallo, 2014 [53]	Three-arm, randomized, open-label	30 females *Group A*: 10 mPEAPol; *Group B*: 10 leuprorelin acetate; *Group C*: 10 ethinylestradiol + drospirenone	Chronic pelvic pain (endometriosis)	NRS	800 + 80 mg/day **mPEAPol** for 6 months	NA	Significant decrease in painful symptoms in all groups regardless of the treatment; improvement in QoL; not interference with woman fertility
Dell’Anna and De Marzi, 2017 [54]	Observational, open-label	17 females	Chronic pelvic pain (endometriosis)	NRS	600 mg/day **umPEA** + 1200 + 120 mg/day **mPEAPol** for 4 months	Analgesics and anti-inflammatory drugs as needed	Significant reduction in pelvic pain, dysmenorrhea, dyschezia, dyspareunia and dysuria; improvement in QoL; not interference with fertility
Stochino Loi et al., 2019 [55]	Single arm, non-randomized, open-label	30 females	Chronic pelvic pain (endometriosis)	VAS	1200 mg/day **umPEA** for 10 days; subsequently 800 + 80 mg/day **mPEAPol** for 80 days	Ketoprofen 80 mg, maximum twice daily	Significant reduction in pelvic pain, dyspareunia, dysmenorrhea, dyschezia; improvement in QoL and psychological well-being; significant reduction in the ketoprofen consumption
Schweiger et al., 2019 [49]	Retrospective, observational	359 (23 males, 336 females)	Fibromyalgia syndrome (FMS)	VAS	1800 mg/day **umPEA** for 10 days; subsequently 1200 mg/day for 20 days; followed by 600 mg/day until the 15th months	Various FMS drug treatments (SSRI, SNRI, GBPs, TCA, BZD, OPI, NSAIDs, MR, ACT)	Significant improvement in pain intensity and QoL

SSRIs: Serotonin selective reuptake inhibitors; SNRIs: Serotonin noradrenaline selective inhibitors, GBPs: Gabapentinoids, TCAs: Tricyclic antidepressants, BZDs: Benzodiazepines, OPIs: Opiates, NSAIDs: Non-steroidal anti-inflammatory drugs; MRs: Muscle relaxants, ACT: acetaminophen; NA: not available, QoL: quality of life.

**Table 2:** Number of patients and outcomes of each study included in the meta-analysis.

Reference	T0 n	T0 NRS/VAS Mean ± SD	T30 n	T30 NRS/VAS Mean ± SD	T60 n	T60 NRS/VAS Mean ± SD
Giugliano et al., 2013 [52] Group A	19	5.8 ± 2.8	19	3.8 ± 2.4	19	2.9 ± 1.7
Giugliano et al., 2013 [52] Group B	28	4.6 ± 2.4	28	2.7 ± 1.7	28	1.6 ± 1.6
Chirchiglia et al., 2018 [50] Group Mild	14	3.5 ± 0.75	14	1.7 ± 0.37	14	0 ± 0
Chirchiglia et al., 2018 [50] Group Moderate	14	5.3 ± 0.37	14	2.6 ± 0.37	14	0 ± 0
Chirchiglia et al., 2018 [50] Group Severe	72	8.7 ± 0.85	72	6.4 ± 1.27	72	4.6 ± 2.46
Paladini et al., 2017 [51]	35	4.3 ± 0.65	35	2.7 ± 0.53	35	1.7 ± 0.65
Scaturro et al., 2020 [42]	120	6.3 ± 1.10	120	3.7 ± 0.99	120	2.0 ± 0.99
Lo Monte et al., 2013 [40]	24	5.1 ± 2.65	23	3.0 ± 2.09	23	1.9 ± 1.65
Di Francesco and Pizzigallo, 2014 [53]	10	5.3 ± 3.63	9	4.8 ± 2.58	9	4.0 ± 2.40
Dell’Anna and De Marzi, 2017 [54]	17	7.8 ± 1.53	16	6.1 ± 2.04	15	4.9 ± 2.56
Stochino Loi et al., 2019 [55]	30	7.2 ± 1.2	30	4.1 ± 0.3	28	2.9 ± 0.2
Schweiger et al., 2019 [49]	359	7.58 ± 1.52	303	6.3 ± 1.98	248	5.9 ± 2.09

n: number of patients; SD: standard deviation.

**Table 3:** Summary of the effects measured.

	T0	T30	T60
NRS/VAS score (Weighted mean ± SEM)	5.93 ± 0.47	3.85 ± 0.47	2.49 ± 0.45
Effect vs. T0 (score)		2.08	3.44
Effect vs. T30 (score)			1.36
Effect vs. previous follow-up (%)		35.1	35.4

SEM: standard error of the mean.
